# Plant-Derived Extracts Feed-Addition and Packaging Type Influence Consumer Sensory Perception of Pork

**DOI:** 10.3390/nu11112652

**Published:** 2019-11-04

**Authors:** Begoña Panea, Guillermo Ripoll

**Affiliations:** 1 Centro de Investigación y Tecnología Agroalimentaria de Aragón (CITA), Avda. Montañana, 930, 50059 Zaragoza, Spain; gripoll@aragon.es; 2 Instituto Agroalimentario de Aragón—IA2 (CITA-Universidad de Zaragoza), C/Miguel Servet, 177, 50013 Zaragoza, Spain

**Keywords:** packaging, exposure time, plant extract, visual appraisal, consumer home-test

## Abstract

This paper investigates whether the combination of the addition of extracts derived from plants (plants derived extracts, PDE) to pork feedstuff and the meat conservation conditions (packaging and time exposure) affect consumers’ perception of pork quality, studied by means of visual appraisal, purchase intention and a home test. The three PDE groups were control, garlic extract and blended oil composed by carvacrol, timol, cynamic aldehide and eugenol extracts. Meat was packed in film, vacuum or modified atmosphere (MAP) packaging. A visual test was designed comprising a four-day storage step followed by a four-day exposure step in a refrigerated island display case. All studied effects influenced visual appraisal scores, being time exposure and packaging effects more noticeable than PDE or pig-sex effects. Meat from MAP scored higher than the rest. Scores decreased as exposure time increased, but this evolution was less perceptible in vacuum packaging and was faster for meat from the garlic group. Only gender affected the visual appraisal scores, with women scoring higher than men. Neither PDE addition nor pig sex affected to purchase intention whereas both exposure time and packaging type did. A maximum of 2 days of exposure would be recommended. In the home-test, meat from male pigs obtained higher scores than meat from female pigs, and none of the consumer-related effects influenced the given scores.

## 1. Introduction

To ensure the healthy, safe and high-quality food demanded by consumers, livestock must be in good sanitary condition. It has been demonstrated that some extracts derived from plants (PDEs) have antimicrobial properties [1,2] and therefore they have been tested to reinforce the control of zoonosis, such as salmonellosis, for example [3]. Nevertheless, animals’ diet modifications can induce meat-quality modifications [4]. If a diet causes changes in the degree of saturation of intramuscular fat, meat could be prone to lipid oxidation during ageing, thus affecting the meat colour [5]. Colour has been reported as one of the most important sensory quality characteristics [6] because it affects freshness perception and consumer’ purchase intention. To guarantee lipid stability, tocopherol and other synthetic antioxidants have been frequently used, but consumers reject these synthetic products; consequently, recent research has been focused on antioxidants from natural resources, including PDEs [7]. Different plants are used for almost all livestock species [8,9,10,11,12,13]. Moreover, since it is widely accepted that diet may affect meat sensory characteristic, there are several studies regarding the possibility of varying sensory profile by means of different plant materials, such as rosemary, oregano, ginger, garlic or chicory [14,15].

Sensory appraisal is a whole consumer experience, including both extrinsic and intrinsic quality cues [16]. Tenderness, juiciness or flavor are the most important intrinsic cues for consumers [17] and as meat is often retail packed in order to lengthen its shelf-life [18], packaging should preserve this intrinsic trait in the finest conditions until consumption [19]. When meat is packed with an oxygen-permeable film, it maintains its attractive red colour, but it is not protected against oxidation or microbial contamination. Vacuum packaging is frequently used in industry because vacuum packaging increases the shelf life of meat by reducing microbial growth [20], but the meat develops a dark-brown colour that is rejected by consumers. Finally, a modified atmosphere (MAP) maintains the meat’s desirable red colour and prevents bacterial growth, but it promotes increased lipid oxidation during storage [21].

Thus, the aim of the paper was to investigate if the combination of the addition of PDE to pork feedstuff and the meat conservation conditions (packaging and time exposure) affect consumers’ perception of pork quality, studied by means of visual appraisal, purchase intention and a home-test.

## 2. Materials and Methods 

### 2.1. Animals and Handling

The procedures used in the trial followed the Spanish guidelines for experimental animal protection [22] and were approved by the Institutional Animal Care and Use Committee of the Research Centre (Procedure number 2011-03). A total of 150 Duroc × (Landrace × Large White) animals that were intended for Protected Geographic Indication (P.G.I.) “Jamón de Teruel” ham production were randomly allotted into three experimental groups: control, garlic and oil. The feed for all animals was a cereal mixture composed of corn, soya, wheat, barley and rapeseed that were given ad libitum. Pigs were housed in 80% slotted floor pens (3.50 m × 3.00 m) in a natural-environment barn and had free access to a pelleted diet and water throughout the trial. The diet was formulated to ensure the requirements of pigs of that age [23]. The compositions of the diets [24,25] are shown in Table 1.

Animals from the control group were fed only this diet, whereas the other two lots were fed with the diet added with each PDE from initiation to slaughter. The garlic group had 1 kg/Tm of a garlic (*Garlic sativum*) complex (Garlicon^®^ Domca, S.A.U., Granada, Spain), resulting from a 25 g/Tm combination of propyl propane thiosulfonate /propyl propane thiosulfonate added to their feed. The oil group feed had 2 kg/Tm of a compound (Repaxol^®^, Molimen, Barcelona) formed by a mixture of carvacrol (from oregano), thymol (from thyme), cinnamic aldehyde (from cinnamon) and eugenol (from clove), which was microencapsulated by a lipidic matrix and added to the feed.

When the animals reached the weight described by P.G.I. “Jamón de Teruel”, they were slaughtered at a slaughterhouse that was authorized by the European Union (EU) (Calamocha, Spain). The pigs were electrically stunned (225 to 380 V/0.5 A for 5 to 6 s), exsanguinated, scalded, skinned, eviscerated, and split down the midline according to standard commercial procedures. Carcasses were kept at 4 °C for 24 h. Then, 24 carcasses from each experimental group (12 barrows and 12 gilts) were randomly selected and subsequently, left loins from the 5th thoracic vertebra to the 6th lumbar vertebra were excised and transported to our laboratory. Once in the laboratory, the m. longissimus thoracis et lumborum were deboned and sliced to obtain the samples described below.

### 2.2. Sample Preparation and Packaging

(a) Visual Appraisal

Since the aim of the experiment was to determine the possibility of lengthening the meat shelf-life, visual test was designed comprising a four-days storage step in different packaging followed by a four-days exposure step in the same packaging.

Then, from each animal, three 2-cm steaks were used for visual appraisal. Steak 1 (FILM) was placed on a polystyrene tray overwrapped with a polyethylene low density (PE-LD) oxygen permeable film (Coimbra Pack, S.L., Spain) without contact with the meat surface and stored until 4th post-mortem day at 4 °C in darkness.

Steak 2 (VACUUM) was vacuum packed (MCOEX material, Coimbra Pack, S.L., Spain), stored until 4th post-mortem day at 4 °C in darkness and thereafter, it was extracted from the vacuum bag and placed on a polystyrene tray overwrapped with a PE-LD oxygen permeable film (Coimbra Pack, S.L., Spain) without contact with the meat surface.

Steak 3 (MAP) was packed under MAP (2:1 gas:meat ratio, commercial atmosphere of 70% O_2_, 30% CO_2_, Praxair España, Spain) with a cover film Cryovac 1825–50, (Cryovac Europe, Barcelona Spain, oxygen permeability of 14.8 cm^3^·m^−2^·24 h^−1^ at 1 atm and a water vapor permeability of 16 g·m^−2^·24 h^−1^) stored until 4th post-mortem day at 4 °C in darkness and, thereafter, it was extracted from the MAP and placed on a polystyrene tray overwrapped with a PE-LD oxygen permeable film (Coimbra Pack, S.L., Spain) without contact with the meat surface.

(b) Home Test

Two 2-cm steaks per animal were vacuum packed (MCOEX material, Coimbra Pack, S.L., Spain), at 1 day of ageing, frozen at −20 °C and destined for the consumers’ home test.

### 2.3. Methodological Procedures

All the consumers participants in both experiments were recruited among students and workers, without relation with the current research, of Aula Dei Campus (Zaragoza, Spain). Personal data such as identification or electronic mail were not required and there was no financial compensation. Participants were clearly informed of the aim of the study and gave implicit consent for research use of the supplied information according to European regulations and the study was conducted in accordance with the Declaration of Helsinki.

(c) Visual Appraisal

Once placed on the polystyrene tray, all the samples were expose for 4 days in a Carrier Multinor 1540/80 refrigerated island display case (Carrier Refrigeración Ibérica SA, Madrid, Spain) with a display area of 1 m^2^ (1.3m × 0.8m) at 0–2 °C, to simulate supermarket conditions. Samples were evaluated by the consumers each day of the storage time, and samples were available to the consumers from 08:00 to 16:00. During this time, samples were moved randomly three times to avoid possible presentation-order, first-order and carry-over effects. The lightning was provided by light-emitting diode (LED) bulbs with a luminous flux of 816 lumen, a color temperature of 4000 K, a color-rendering index >80 and a standard deviation color matching equal to MacAdam ellipses. The illuminance on the surface of the chops was approximately 1300 lx, ensuring the minimum level of illuminance in areas with high visual requirements.

Consumers was provided with a form on which they were asked about their gender and age. Regarding the steaks, the consumers were asked to evaluate the appearance of 5 samples using a continuous 10-point scale, from 1 (very bad) to 10 (very good). Additionally, they were asked about their purchase intention (yes/no).

(d) Home Test

Each consumer had a pack including 2 loin portions, one each from a different combination pig-sex*PDE. Test were performed following a complete balanced design. Samples of the packet were identified by a three-figure random number and they were accompanied by an evaluation questionnaire. Consumers were not given any information about the steaks other than the species and accurate guidelines for storage and cooking were specified on the questionnaire. Consumers evaluated each sample for preference for taste, juiciness and tenderness, using a continuous 10-point scale (from 1 = dislike very much, to 10 = like very much). Consumers were also asked their gender, age, general liking for meat (high, medium, low) and weekly meat consumption (1–2; 3–6; daily).

### 2.4. Statistics

Statistical analyses were performed using XLStat 17.03 software. Consumers were categorized into 4 categories of age.

Visual appraisal score was analyzed using two independent analysis of variance (ANOVA) procedures: the first with PDE, pig sex, exposure time and type of packaging as main effects; the second ANOVA with the consumer’s gender and age as main effects. Means for visual appraisal scores were calculated. The Duncan test was used to compare means, and the level of significance was *p* < 0.05. Crosstabs for studied effects were carried out and the influence of the effects on purchase intention was investigated by the chi-square test.

For the consumers’ home test, preference for juiciness, taste and tenderness scores were mean-centered. In this way, the effect of the use of the scale was eliminated. Then, we used two ANOVA procedures, the first with PDE and pig sex effects and the second with consumer’s gender, consumer’ age, liking for meat and meat-consumption frequency as main effects. The means for the corrected scores in function of significant effects were calculated. The Duncan test was used to compare means, and the level of significance was *p* < 0.05. Crosstabs for studied effects were carried out and differences for scores were investigated by the chi-square test.

## 3. Results

### 3.1. Visual Appraisal Scores and Purchase Intention

#### 3.1.1. Effect of Extracts Derived from Plants (PDE), Pig Sex, Exposure Time and Packaging Type

**Figure 1:**
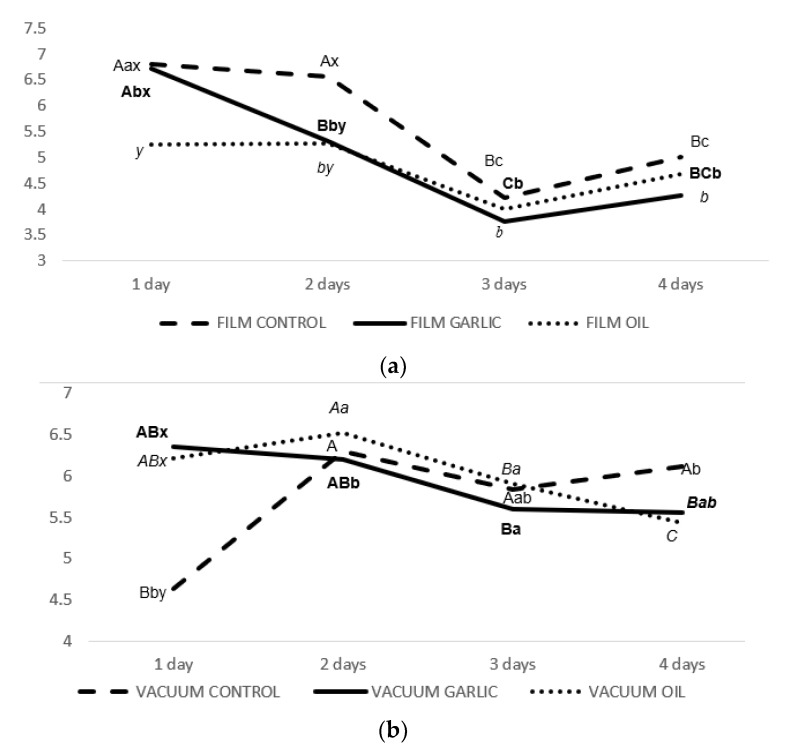

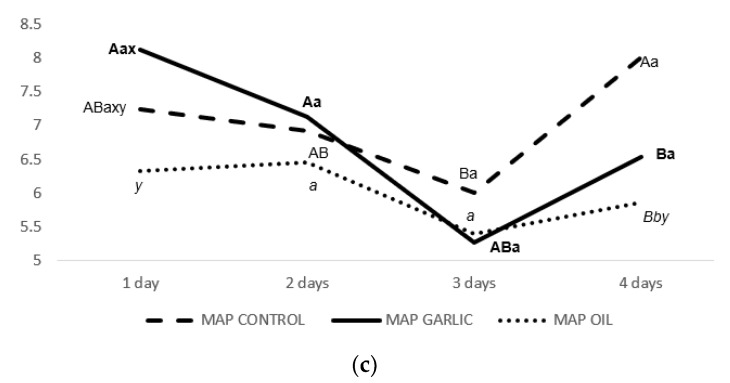
Visual appraisal scores of pork meat in function of PDE feed-addition, exposure time and packaging type. (**a**) Film packaging; (**b**) vacuum packaging; (**c**) MAP (modified atmosphere) packaging). a,b. Different letters means statistical differences (*p* < 0.05) between packaging types inside PDE group and exposure time. A,B. Different letters means statistical differences (*p* < 0.05) between exposure time inside PDE group and packaging type. x,y. Different letters mean statistical differences (*p* < 0.05) between PDE groups inside packaging type and exposure time. Bold letters-garlic group. *Italics*-oil group.

A total of 179 people filled in the survey. All studied effects influenced significantly the visual appraisal scores (Table 2), being time exposure and packaging effects more noticeable than PDE or pig sex effects. Both time exposure and packaging type presented significant interactions between them and with PDE, whereas pig sex did not. None of the triple or quadruple interactions were significant. Then, we obviated sex effect and studied visual appraisal scores in function of PDE group, exposure time and packaging type, which were shown in Figure 1.

Global means for PDE groups were 6.42 for control group, 6.02 for garlic group and 5.73 for oil group. Global mean for males was 6.27 whereas global mean for females was 5.95.

In general, visual appraisal scores decreased as exposure time increased. Nevertheless, this evolution was less perceptible in VACUUM than in FILM or MAP. The effect of PDE disappeared from the 3rd exposure-day onward in FILM. At 1 day of exposure, the oil group obtained lower scores than the rest. The decrease for scores were faster for meat from garlic group than for meat from control group. Regarding VACUUM, meat without PDE shown the lowest scores at 1st day of exposure but from the second day of exposure onward no differences were found between the three different PDE groups. Finally, within MAP, the effect of PDE was noticeable, with oil group showing the lowest scores at any exposure time. In general, MAP scored higher than VACUUM or FILM.

**Figure 2:**
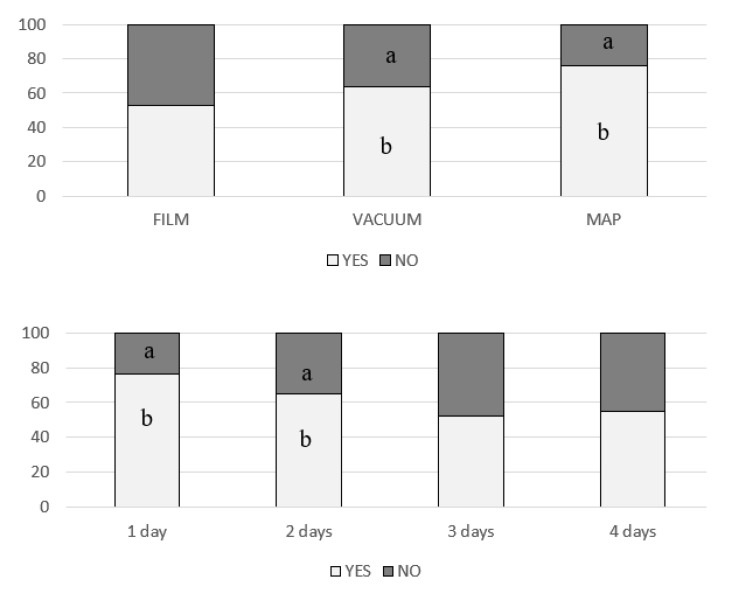
Percentages of purchase intention in function of packaging type and exposure time. a,b Different letters means significant *p* value (*p* < 0.05) for X^2^ test inside a packaging type or inside a exposure time.

Regarding purchase intention, neither PDE addition (*p* = 0.124) nor pig sex (*p* = 0.149) affected purchase intention whereas both exposure time and packaging type did (*p* < 0.000). Figure 2 shows percentages of purchase intention in function of packaging type and exposure time. No differences between YES percentages and NO percentages were found for film whereas percentages of YES were higher than percentages of NO in vacuum packaging or in MAP packaging. Frequency of YES were higher than No frequencies at 1st exposure-day and at 2nd exposure-day whereas no differences between YES percentages and NO percentages were found at 3rd or at 4th exposure-day. Therefore, a maximum of two days of exposure is recommended.

#### 3.1.2. Effect of Consumer Profile

Females (Table 3) represented 58% of respondents and by ages, 7.3% were less than 25 years old, 30.5% were from 26 to 40 years old, and 48.4% were from 41 to 55 years old.

Only gender affected the visual appraisal scores (*p* < 0.0001) and no significant interactions were found between consumer’s gender and consumer’ age (*p* = 0.071). Table 4 shown the means and standard error for visual appraisal scores. In general, women gave higher scores (6.3 on average) than men (5.8 on average). Crosstabs shown that men scored 4 or 5 more frequently than expected whereas women scored 8 or 10 more frequently than expected (X^2^ < 0.000). Men gave the highest scores to meat from the control group whereas women gave the lowest scores to meat from the oil group. All the respondents scored the meat packed in MAP packaging higher than meat packed in film or vacuum packaging, but women distinguished between film packaging and vacuum packaging whereas men did not. Similarly, women clearly penalized meat exposed for 3 or 4 days whereas men only discriminated clearly meat with 1 day of exposure.

Consumers’ gender did not affect purchase intention (*p* > 0.05) whereas consumer’ age showed a tendency (*p* = 0.052). Independently of the consumer age group (Table 5), percentages of YES (I’d buy it) were higher than percentages of NO. Crosstabs between consumers’ ages and PDE, pig sex, packaging type or exposure time shown than people aged ≤25 preferred meat from 1 day-exposure time more frequently than expected (*p* = 0.042). Likewise, people aged 26–40 chosen film packaging less frequently than expected (*p* < 0.000) and preferred meat from 1 day-exposure time more frequently than expected (*p* = 0.025). People aged 41–55 also preferred meat from 1 day-exposure time (*p* = 0.018) and they chose meat from the control group instead of meat from the oil group (*p* = 0.004). Finally, people aged >55 chose film packaging less frequently than expected (*p* = 0.008) and preferred meat from 1 day-exposure time (*p* = 0.007) but they depreciated meat from garlic group and valorised meat from oil group (*p* = 0.016). A resume of these preferences is shown in Table 6.

### 3.2. Consumers’ Home Test

#### Effect of PDE and Pig Sex

A total of 72 people filled the enquiry (Table 7). Sample was equally spread by gender and age. Most people like meat (73.6%) being higher the percentage for men (89.7%) than for women (57.6%). Independent of the gender, higher meat-frequency consumption is 3–6 times a week

Table 8 shows the *p* values for PDE addition and pig-sex effects in a pork-meat consumer’s home-test. PDE addition affected both taste (*p* = 0.012) and juiciness (*p* = 0.047) whereas sex only affected taste (*p* = 0.012). The interaction between effects was significant for both juiciness (*p* = 0.001) and tenderness (*p* = 0.002).

**Figure 3:**
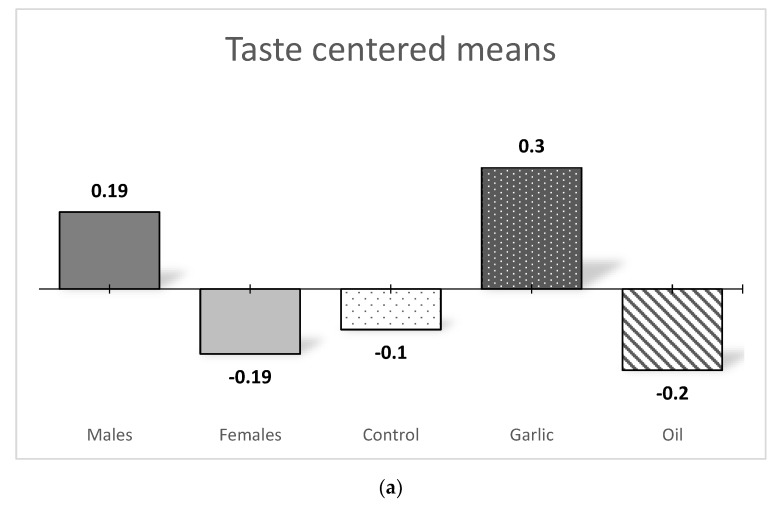

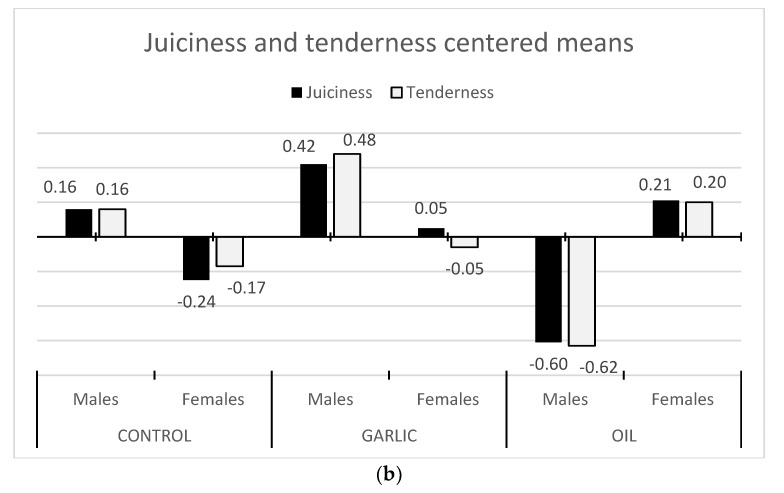
The centered means for sensory variables in a pork-meat consumers home test in function of pig sex and PDE addition. (**a**) centered mens for taste in function of pig-sex or PDE addition. (**b**) centered means for juiciness and tenderness in function of pig sex and PDE addition.

Figure 3 shows centered means for the taste variable as well as the *p* values for PDE addition and pig-sex effects. In this picture, when mean-centered value was positive, scores for the item were higher than the global mean. Meat from male pigs obtained higher scores than meat from female pigs. Regarding the PDE addition effect, garlic group obtained higher scores than the other two groups, with no statistical differences between them.

### 3.3. Effect of Consumer Profile

None of the consumer-related effects (gender, age, linking for meat or frequency of meat consumption) influenced the given scores (*p* > 0.05).

## 4. Discussion

### 4.1. Visual Scores and Purchase Intention

There are many studies about influence of the PDE on meat quality and most of them have focused on TBARs (thiobarbituric acid reactive substances), colour or flavour [15,26,27,28,29] but from the knowledge of the authors, almost none of them regard the effect on visual appraisal or on purchase intention. Ripoll et al. in a study with lambs reared in 4 different feed regimes found there was a significant relationship between feeding system and visual appraisal scores of M. rectus abdominis because of differences in colour [30]. This conclusion can explain the effect found for PDE addition on visual scores, since as reported in a previous paper with the same animal material used in the current experiment [31] differences were found in meat colour between animal groups. Colour was defined such as the most important factor influencing consumer perception, and more important than other factors as marbling or sample shape [32], because colour is used by consumer as an indicator of spoilage [33]. 

Borgogno et al. found, in packed goat meat, that the liking scores decreased significantly from the first day to the third day [34]. In a study with several bones, Grobbel et al. found an interaction bone–packaging–display time for visual appraisal since colour changes and they concluded that the darkening was more extensive for sample packaged in PVC (polyvinylchloride) and high-oxygen MAP than for those in ultra-low oxygen MAP [35]. In the current experiment, and independently on the packaging and PDE addition effect, colour hue values decreased from the 1st to the 4th day (61.5 and 58.5, respectively), which could to explain the effect of exposure time on visual scores. In current experiment, meat packed in MAP had in general higher visual scores than meat packed in vacuum or overwrapped and hue values for MAP were higher in MAP than in film or vacuum packaging at both 1st (63. 7 in MAP, 57.8 in film and 63.1 in vacuum) and 4th days of exposure (61.9 in MAP, 55.5 in film and 58.2 in vacuum). This result disagreed with those reported by other authors [36] who described that meat packed in PVC is preferred to meat packed in other packaging. Nevertheless, Carpenter et al. found a positive correlation between visual scores and purchase intention [36], which is in agreement with current results, since the higher percentages for purchase likelihood were found in MAP (76.2%; Table 3).

In the current experiment, a consumer’s gender influenced visual appraisal scores, according with Jiang et al. who concluded that gender affects the emotion relevance to food in specific way [37]. Femininity and masculinity have been associated with the type of food that individuals eat [38]. It has been reported that females show higher preference for white meat because females are often suspicious about red meat and express less meat-eating satisfaction and this finding is much more intense in young females, whose expectation about red meat is linked to negative attitudes [32,39,40]. Consumer age is often considered in consumers studies and there are many studies focused on how socio-demographic variables influenced the likelihood of eating meat [41,42] the influence of extrinsic cues, such as health value or risk perception [43] or on consumer tasting perception of cooked meat [33,41,44] but scarce literature is available regarding age differences on visual appraisal and, when it exists, the clustering of consumers is often made based on marbling or colour [45,46,47]. For example, Brewer et al., in a visual appraisal experiment with pork of different marbling degree concluded that highly marbled chops appeared lighter coloured, had less acceptable appearance, and were less likely to be purchased than leaner chops [48]. Current results show that a consumer’s age modified the visual perception, that is, the quality expectation. Most of the consumers expressed that film was the least preferable packaging and also, they concurred that time-exposure depreciate visual appearance of the meat, but age-related differences were found for the PDE group who chose this “as the most preferably” from a visual point of view. Lahucky et al. reported that addition of oregano prevents to oxidation and then, protects to meat color, which can to explain why oil group was chosen by older consumers [49].

### 4.2. Consumer Home Test

Some authors have reported that the eating quality of the cooked pork was unaffected by the plant extracts in diet [15,49] whereas other authors reported a slight effect of plant addition on sensory attributes [28], being juiciness higher in control pork than in supplemented meat and without differences in tenderness, which agree with present results [26]. As in the present study, meat from animals used by Rossi et al. [26] had no differences on chemical parameters which in turn make difficult the explanation of the results. In the current experiment, no differences in intramuscular fat content were found between groups and hence, no great differences in sensory characteristics were expected a priori but in fact we have a significant interaction between PDE group and pig sex (*p* = 0.001). Although it is generally accepted that an increased level of intramuscular fat has a positive influence on the sensory quality of pig meat, literature reveals contradictory results. For example, Flores et al. found a close relationship between intramuscular fat content and juiciness although this was lower with hardness [50] but Fernandez et al. found that in the case of Duroc crosses, such as was the case of the animals of the current experiment, sensory characteristics were only slightly affected by intramuscular fat level [51]. Several studies have proposed that a minimum of 2% of intramuscular fat is necessary for sensory acceptance of pork [52] but as summarized in Font i Furniols et al., there was not a consensus in literature about this percentage. The effect of intramuscular fat on consumer preferences has been associated with skatole concentration of fat tissue because skatole is responsible for boar taint [45]. In this sense, Leong et al. reported that there is an increase in skatole and indole concentrations in the fat tissue when garlic essential oil was included the diet [53] but the present result has shown that the garlic group obtained higher taste scores than control or oil groups.

Concerning pig sex, D’Souza et al. found that meat from barrows was more tender than meat from females, whereas the sex of the pigs did not influence either juiciness or overall acceptability of the pork [54]. Also, Elsbernd et al. reported no pig-sex effect on juiciness, tenderness, or chewiness of pork [55]. Nevertheless, our results have shown that meat presented a meat with a better taste and, in general, was more tender and more juicy. Font et al. shown that literature is not consistent in the effect of boar taint on meat acceptability and some studies show no differences in the acceptability of meat from entire male and female or castrates whereas others do [56].

Regarding consumers’ profile, Ngapo et al. in a consumer study carried out in several countries, found that most Spanish consumers like pork (90% of respondents), the taste is the most important reason to eat it (81% of respondents), consumer eat pork once a week (67%) and they think that the pork they buy is always or almost always of good quality (90%) [40]. These results agree with those reported by other studies on Spanish consumers [56,57].

Some authors reported that the acceptability of the pig meat depends on the gender and age of consumers, in disagreement with our results [56]. Mathews et al. reported that women were more critical than men and that, in general, the oldest group of consumers had the lowest percentage of dislike scores, both for both flavor and odor [58]. In the same way, it has been reported that consumers over 30 and especially the middle-aged group (31–50) place more emphasis on tenderness than younger consumers [44]. 

Also, some authors have argued that consumers preferences depend on several factors, such as tradition, religion, age, education, gender, income, etc. [59,60] but other authors [16,61] have concluded that socio-demographic variables as well as some life-style related variables, such as frequency of consumption, were less significant than intrinsic cues in the consumer global appraisal.

## 5. Conclusions

All studied effects influenced visual appraisal scores, with time exposure and packaging effects being more noticeable than PDE or pig-sex effects. Scores decreased as exposure time increased, and the decrease was faster for meat from the garlic group than for meat from the control group. Meat from MAP scored higher than meat from vacuum packaging or from film packaging and a maximum of 2 days of exposure would be recommended. Neither PDE addition nor pig sex affected purchase intention whereas both exposure time and packaging type did. In the home test, meat from male pigs obtained higher scores than meat from female pigs and the garlic group obtained higher scores than the rest.

None of the consumer-related effects (gender, age, linking for meat or frequency of meat consumption) influenced the given scores in the home test and only gender affected visual appraisal scores.

Therefore, consumer profile was not an important factor but time exposure and packaging type definitely affect consumer appraisal.

## Figures and Tables

**Table 1:** Composition of basic feedstuff that is used in the experiment.

	Initiation from 20 to 45 kg	Growth from 45–75 kg	Growth from 75 kg to Slaughter
	**Ingredients composition**		
Corn (%)	10.0	-	-
Sunflower undercoated (%)	-	3.7	-
Sunflower 28% (%)	-	-	2.0
Soya 44% (%)	15.9	7.6	1.8
Rapeseed (%)	4.0	10	12.0
Wheat (%)	10.0	15	16.0
Barley (%)	53.7	61	60.6
	**Proximate composition**		
Dry matter (%)	89.2	89.5	89.4
Crude protein (%)	16.0	15.0	14.0
Crude fibre (%)	4.6	5.0	5.5
Non digestible fibre (%)	16.8	18.3	19.7

**Table 2:** Values of *p* for studied effects (plant extract addition, pig sex, exposure time and packaging type) on consumer visual appraisal scores of pork meat.

Effect	*p* Value
PDE addition (D)	0.015
Pig sex (S)	0.001
Exposure time (T)	<0.000
Packaging type (P)	<0.000
D × S	0.590
D × T	0.005
D × P	0.011
S × T	0.293
S × P	0.265
T × P	<0.000

PDE—Plant derived extracts.

**Table 3:** Consumer profile for visual appraisal and purchase intention tests.

Consumers’ Age Groups	Men	Women
≤25 years (%)	9.4	5.7
26–40 years (%)	26.6	33.3
41–55 years (%)	36.0	57.2
>55 years (%)	28.0	3.8

**Table 4:** The means and standard error of studied effects (consumer’ gender and age) on visual appraisal scores of pork meat.

	Treatment	Men	Women
PDE group	Control	6.1 a	6.6 a
	Garlic	5.6 b	6.3 a
	Oil	5.6 b	5.8 b
Packaging type	Film	5.5 b	5.8 c
	Vacuum	5.7 b	6.3 b
	MAP	6.5 a	7.0 a
Exposure time	1 day	6.5 a	6.9 a
	2 days	5.9 b	6.5 a
	3 days	5.1 c	5.6 b
	4 days	5.5 bc	5.9 b
Global mean		5.8	6.3
s.e.		0.09	0.08

a,b. Different letters in a column means significant *p* value (*p* < 0.05) inside PDE groups, packaging types or exposure times. MAP—modified atmosphere packaging.

**Table 5:** The percentages for purchase intention of pork from in function of consumer’ age.

Consumer’ Age	Purchase Intention	
	Yes (%)	No (%)
≤25	70.8 a	29.2 b
26–40	61.9 a	38.1 b
41–55	61.2 a	38.8 b
>55	73.2 a	26.8 b

a,b. Different letters in a row means significant differences between percentages (*p* < 0.05).

**Table 6:** Resume of consumer preferences derived from crosstabs between consumer’ age and PDE, pig-sex, packaging type or exposure time.

Consumers’ Age	Frequency < Than Expected	Frequency > Than Expected
≤25	Film packaging	Garlic group 1 day-exposure meat
26–40	Film packaging 3 days-exposure meat	1 day-exposure meat
41–55	Oil group	Control group 1 day-exposure meat
>55	Film packaging 3 days-exposure meat Garlic group	Oil group 1 day-exposure meat

**Table 7:** Consumers’ profile for the home test. Percentages of valid responses.

Survey Question	Consumers’ Age Groups	Men	Women
	≤25	6.9	6.1
Age	26–40	55.2	54.2
	41–55	24.1	24.2
	>55	13.8	15.2
Do you like meat?	Like very much	89.7 a	57.6 b
	Neither like nor dislike	10.3 b	42.4 a
	Not very much	0.0	0.0
How often a week do you eat meat?	1 or 2	17.2	27.3
	From 3 to 6	75.9	63.6
	Daily	6.9	9.1

a, b letters means statistical differences between genders in a X^2^ test.

**Table 8:** The *p* values for PDE addition and pig-sex effects in a pork-meat consumers home test.

Effect	Taste	Juiciness	Tenderness
PDE (D)	0.012	0.047	0.107
Sex (S)	0.012	0.930	0.928
D × S	0.103	0.001	0.002
